# Direct 3D Printing of Recycled PET/PP Granules by Shear Screw Extrusion

**DOI:** 10.3390/polym15244620

**Published:** 2023-12-05

**Authors:** Dashan Mi, Jie Zhang, Xianqing Zhou, Xinhe Zhang, Shikui Jia, Haiqing Bai

**Affiliations:** 1 School of Mechanical Engineering, Shaanxi University of Technology, Hanzhong 723001, China; 2 State Key Laboratory of Polymer Materials Engineering, College of Polymer Science and Engineering, Sichuan University, Chengdu 610065, China; 3 AVIC Shaanxi Huayan Aero-Instrument Co., Ltd., Hanzhong 723000, China; 4 School of Materials Science and Engineering, Shaanxi University of Technology, Hanzhong 723001, China

**Keywords:** recycled PP, recycled PET, shear screw printer, 3D printer

## Abstract

This article introduces a one-step extrusion-based fused deposition modeling (FDM) approach for the challenging separation of polypropylene (PP) and polyethylene terephthalate (PET) during recycling. A shear screw printer (SSP) with shear elements was designed, and it was compared to a conventional single-screw printer (CSP) to investigate the differences in print stability, degradation levels, tensile performance, molecular orientation, and crystallization when preparing recycled PP and recycled PET blends. Although the retention effect of the SSP screw slightly increases the degradation of the blended rPP/rPET, the strong shear (2.6 × 10^4^ s^−1^) applied near the extrusion exit improves the blending efficiency. The SSP also enhances molecular orientation, modulus of the parts, and reduces performance fluctuations. Additionally, the SSP has the potential to simplify the recycling process, enabling the transformation of blended recycled materials into products with just one melt process.

## 1. Introduction

The global reliance on plastic has grown due to its lightweight nature, chemical inertness, ease of molding, excellent insulating properties, low thermal conductivity, and impressive transparency. The global plastics production for 2021 was 430.7 Mt and China alone accounts for 28% of global resin production [1,2]. Among these polymers, polyethylene (PE) stands out as the most widely used, accounting for 29.7% of the total, closely trailed by polypropylene (PP) at 17.4% [3]. After the use of PE and PP plastics, the pervasive impact of these millions of tons of plastic pollution in oceans and landfills affects everyone. Collectively, PE and PP are responsible for nearly half of the total plastic waste generated.

Recycling offers the potential to reduce the overall environmental impact across a product’s life cycle. Multiple approaches are available for recycling polyolefin waste, encompassing mechanical recycling, pyrolysis, gasification, depolymerization, and other methods [4,5]. Among these methods, mechanical recycling stands out as the most suitable technique for the cost-effective production of recycled plastics, particularly for polymer composite manufacturing.

One of the challenges in polymer recycling involves the necessity of segregating different types of plastic waste. Sometimes, it becomes essential to recycle them as mixed polyolefins. For instance, it is common to encounter PP waste that is contaminated with small quantities of PET and vice versa [6]. A prime example is plastic water bottles, which feature a PET body and PP caps. Consequently, mechanically recycling immiscible mixtures can result in reduced mechanical properties due to poor interfacial compatibility and a lack of adhesion between dissimilar polymers [7]. This issue is particularly pronounced in the case of blends involving polar plastics, such as PET, and non-polar plastics like PP, making it challenging to recycle them into products suitable for high-quality applications [8]. Numerous researchers have detailed the optimization of such blends by employing compatibilizing copolymers like SEBS or grafted PP, where one component can interact with each phase, resulting in mechanical properties that meet the required commercial standards [9,10].

Additive manufacturing (AM), commonly referred to as 3D printing, is experiencing growing utilization in significant industrial sectors, including automotive, aerospace, and healthcare. Among various AM techniques, fused deposition modeling (FDM), also known as material extrusion printing, stands out as one of the most extensively adopted methods for rapid prototyping and designing composite components due to its simplicity, high speed, and cost-effectiveness [11].

The FDM technology can be used for the preparation of recycled plastics, and its process can be roughly divided into three steps. The first step involves using a twin-screw extruder to blend and pelletize the recycled materials to achieve a more uniform phase dispersion. The second step uses a single-screw extruder and stretching equipment to produce filaments with a certain diameter. The third step involves the preparation of samples, where a filament of material is fed into the melting system of a 3D printer via a pinch roller [12,13].

Traditional FDM-based methods for preparing recycled plastics typically involve three cycles of heating, melting, and cooling. In contrast, screw FDM enables the direct use of plastic pellets for printing. With the utilization of screw FDM, recycled materials are expected to undergo only one melting–cooling process. This reduction streamlines the procedure, reduces energy consumption required for heating, and alleviates thermal degradation of the recycled materials. Moreover, screw FDM has the capability to process a broader range of materials that may not be available in filament form or possess the required stiffness and strength for conventional FDM [14].

Consequently, the direct use of plastic pellets for printing offers numerous advantages, leading to the development of various screw FDM technologies. For example, Liu et al. [14] developed a large-scale double-stage-screw 3D printer, capable of efficiently producing large plastic molds and products at a low cost and high speed. Joaquim et al. [15] outlined a design workflow for a new print head based on co-rotating twin-screw extrusion. Tsan et al. [16] designed a screw printer with a pellet-feeding mechanism, allowing the printing of high-viscosity PEEK. Netto et al. [17] discussed more than 30 types of screw-assisted printers and aimed to provide a systematic design approach.

In this research, we developed both a conventional single-screw printer (CSP) and a high-shear single-screw printer (SSP) for 3D printing of recycled polypropylene (rPP) and recycled polyethylene terephthalate (rPET). A comparative analysis was conducted, focusing on printing accuracy, mechanical performance of printed components, and the material degradation between CSP and SSP. Currently, research on 3D printing with recycled materials often employs traditional methods such as blending, filament extrusion, and printing, while limited attention is given to one-step printing. This study explores the influence of screw shearing in 3D printing using recycled materials, proposing an approach that simplifies the recycling process through a single heating step. The research aims to streamline the recycling process of rPP/rPET, saving heating energy. Additionally, it designs an FDM extrusion screw suitable for blending recycled materials, aiming to enhance dimensional stability during printing and improve the dispersion of the blended material.

## 2. Experimental Section

### 2.1. Mechanics Design of the Screw Extruder

**Figure 1:**
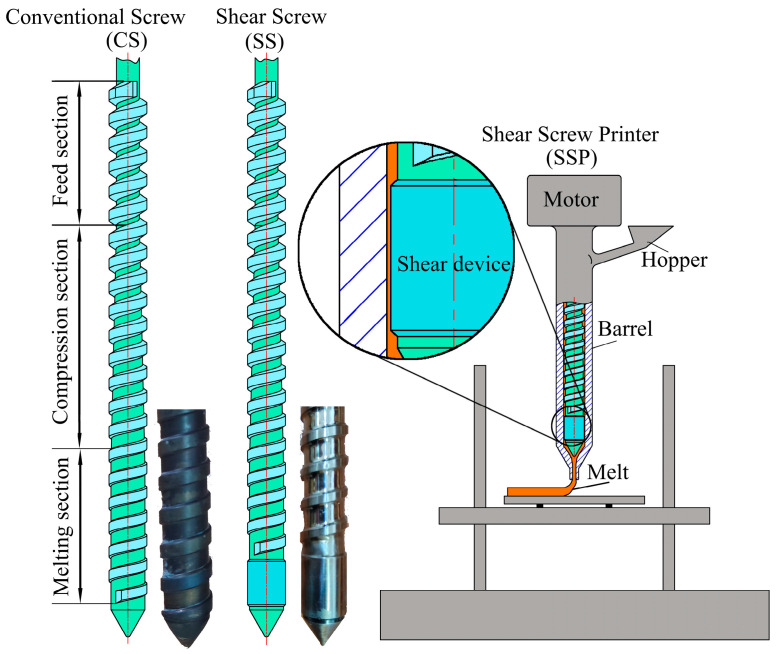
The schematical of convention screw (CS), shear screw (SS), and shear screw printer (SSP).

Figure 1 illustrates the schematical of the extruder printer, and Table 1 provides the mechanical design values for the screw. The screw configuration is divided into three sections: material feeding, compression, and metering zones. The screw used in this study is based on a conventional screw extrusion machine. The chosen aspect ratio (L/D) is 15:1 with a screw diameter (D) of 16 mm. The lengths of the feeding, compression, and metering zones are approximately 4D, 6D, and 5D, respectively. Other specifications include a fixed pitch (t = 10 mm), clearance to the barrel (λ = 75 μm), and flight width (s = 4 mm). The compression ratio (ε) is calculated using the formula [16]:

(1) $$\varepsilon =\frac{h_{F}(t-s)(D-h_{F})}{h_{M}(t-s)(D-h_{M})}$$

where h_F_ is the feeding zone depth and h_M_ is the metering zone depth. Setting h_F_ = 3.1 mm and h_M_ = 0.8 mm yields a designed value of ε as 3.29.

In the feed and compression sections, both conventional screw (CS) and shear screw (SS) have similar structures. However, the melting section of SS incorporates a shear device to impart shear capabilities. As this shear device is located at the extrusion end, it is expected to retain the shear effect on the extruded melt. The shear rate can be calculated using [18]:

(2) $$\dot{\gamma }=\frac{2\pi D_{A}N}{2\delta }$$

with the external diameter of the actual screw (D_A_ = 16 mm), the rotation speed of the screw (N = 39 rpm) and the clearance between the screw and the barrel (*δ* = 0.075 mm); the shear rate was calculated to be 2.6 × 10^4^ s^−1^. Subsequently, CS and SS were separately incorporated into our self-developed printer, resulting in the creation of a conventional screw printer (CSP) and a shear screw printer (SSP).

### 2.2. Materials and Sample Preparation

Both recycled PP (rPP) and recycled PET (rPET) were purchased from Blue Planet Biodegradable Materials (Dongguan) Co., Dongguan, China. The rPET underwent a 5 h drying process in an oven at 120 °C. Poly(propylene-g-maleic anhydride) copolymer (PP-g-Ma) with a graft content of 1.2 wt.% is a commercially available product supplied by Guangzhou Lushan Chemical Materials Company, Guangzhou, China.

After extrusion, the melt is cooled in the air and then layered to print the tensile bars. The mixture is incrementally added to the hopper after mechanical blending. The content of r-PET and PP-g-Ma is 30 wt.% and 10 wt.%, respectively. All samples are labeled based on the polymer component and printer as Table 2 shows.

To prevent sample warping during the printing process, the bed temperature was set at a higher value of 120 °C. Additionally, for a comparative assessment of the shear effects of CSP and SSP on the blend, both were subjected to the same extrusion temperature. Through multiple experiments, it was determined that an extruder temperature of 280 °C adequately accommodates both CSP and SSP, ensuring the formation of a stable extrusion melt. Tensile bars were printed with a 0° infill orientation. The nozzle diameter is 0.3 mm, and the layer thickness is 0.5 mm. Due to the pressure drop caused by the shear device in the SS, the SSP necessitates a higher screw speed to achieve the same extrusion volume as the CSP. The higher screw speed in the SSP further enhances its shear capability. During the printing process, the extruder speed for CSP and SSP is 11.7 and 39 RPM, respectively.

### 2.3. Sample Testing

#### 2.3.1. Measurement of Mechanical Properties

**Figure 2:**
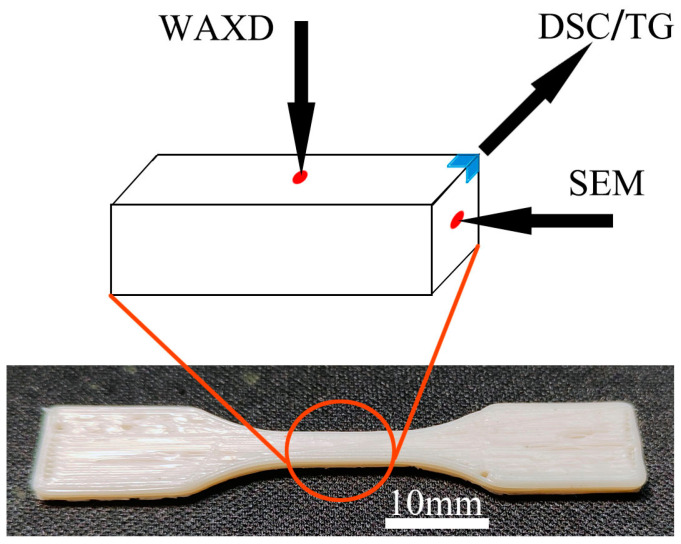
The printed tensile bar and the location of different tests.

Figure 2 displays the printed tensile bar and indicates the test location. Tensile testing was performed at room temperature (26 °C) using an electro-universal testing machine (GOTECH-20KN, GOTECH Testing Machines Inc. Dongguan, China) with a cross-head speed of 5 mm/min according to GB/T 1040 [19]. The force direction aligned with the printing direction and property values were averaged from five samples.

#### 2.3.2. Two-Dimensional Wide-Angle X-ray Diffraction (2D-WAXD)

The 2D-WAXD experiment was performed using the HomeLab system (Rigaku, Tokyo, Japan). Specimens were cut from the bars’ centers, and X-ray imaging was performed after penetrating the samples. The rectangular beam had dimensions of 100 × 100 μm^2^, and the wavelength of the light was 0.154 nm. The full-width at half maximum (FWHM) was a suitable characteristic to measure crystalline phase orientation. The degree of orientation was quantified using the relative orientation parameter f [20]:

(3) $$f=\frac{180{}^{\circ}-FWHM}{180{}^{\circ}}\in (0,\;1)$$

The value of f ranged from 0 to 1, with higher values indicating a transition from random crystal reflection to full alignment along the flow direction.

#### 2.3.3. Morphological Characteristics

The morphologies of recycled PP, PP/PET, and PP/PET/Ma were examined using scanning electron microscopy (SEM). The specimens were gold-sputtered after being stretched to yield. The fracture surface of each sample was characterized using a ZEISS Gemini 300 SEM from Oberkochen, Germany.

#### 2.3.4. Thermal Properties (Differential Scanning Calorimetry (DSC) and Thermogravimetric Analysis (TGA))

The melting behavior of the samples was assessed using a TA Discovery DSC 250 (TA Instruments Co., New Castle, DE, USA) under a nitrogen atmosphere. The heating and cooling rate was set at 10 °C/min.

The percent crystallinity, X_c_, was determined by the following equation:

(4) $$X_{c}=\frac{{∆H}_{m}-{∆H}_{cc}}{{∆H}_{100}}\times 100\%$$

where ${∆H}_{m}$ is the enthalpy of melting, ${∆H}_{cc}$ is the enthalpy of cold crystallization, ${∆H}_{100}$ is the theoretical enthalpy of melting for a 100% crystalline PP, which is for 207 J/g [9].

The thermal stability of recycled PP and composites was analyzed using a thermogravimetric analyzer (HITACHI STA200, Tokyo, Japan) in a nitrogen environment, with a heating rate of 10 °C/min within a temperature range of 50–800 °C.

## 3. Results and Discussion

**Figure 3:**
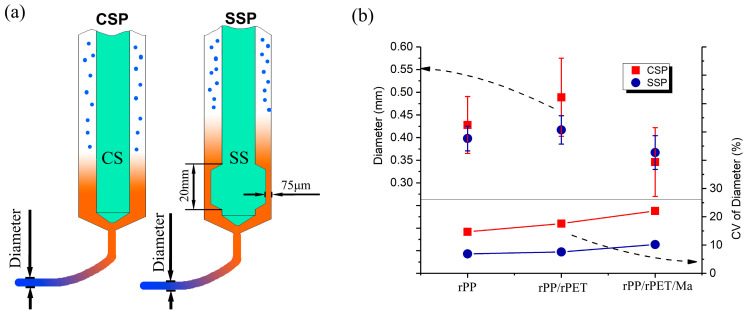
(**a**) Extrusion diagram of CSP and SSP and (**b**) diameter of extruded filament and the CV of diameter.

The stability of the extruded filament diameter plays a crucial role in ensuring the dimensional accuracy of FDM parts. In comparison to CSP, SSP is capable of achieving a more consistent extruded filament diameter. As Figure 3a shows, SSP features a 20 mm long shear device with only a 75 μm gap between the shear device and the barrel wall. This shear device acts as a flow restriction, causing a significant pressure drop as the melt passes through it. Consequently, an increase in screw speed is necessary during SSP operation, and more molten polymer is blocked by the shear device.

In the case of industrial single-screw extruders, the pressure drop introduced by the shear device can hamper production efficiency and is not commonly employed. However, for small-scale single-screw FDM, where the required extrusion volume is not high, the pressure drop resulting from the shear device has no adverse effect on printing efficiency. Instead, it provides advantages, including improved fluid stability, better plasticization, and enhanced blending effects.

Figure 3b is divided into two sections, with the upper part showing the average extrusion diameters of CSP and SSP, and the lower part illustrating the magnitude of diameter fluctuations. As Figure 3b demonstrates, when operating at speeds of 39 rpm for SSP and 11.7 rpm for CSP, the extruded filament diameters are quite similar. For example, extruded rPP exhibits diameters of 0.425 mm and 0.450 mm through CSP and SSP, respectively. When rPET and PP-g-Ma are added, changes in viscosity and other parameters result in filament diameter fluctuations within the range of 0.35 mm to 0.50 mm. The average diameter difference between CSP and SSP for the same components is within 16%.

Nevertheless, CSP’s inadequate blending leads to non-uniformity at the exit, causing fluctuations in melt viscosity and, consequently, larger variations in filament diameter, especially in the case of rPP/rPET and rPP/rPET/Ma. For example, the diameter standard deviation after SSP extrusion for rPP/rPET is 0.03 mm, while CSP’s extrusion diameter standard deviation is 0.09 mm. To better measure diameter size fluctuations, the coefficient of variation (CV) was utilized. For CSP, the CV is higher than 10%, and the CV for rPP/rPET and rPP/rPET/Ma reaches 18% and 22%, respectively. In contrast, for SSP, the CV for rPP/rPET and rPP/rPET/Ma is only 8% and 10%, respectively. This is likely attributed to the higher shear screw speed and shear stress of SSP, improving blending effects, and resulting in stable melt viscosity.

**Figure 4:**
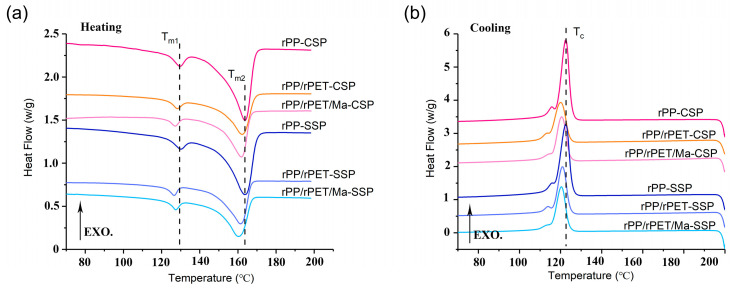
DSC curves of recycled PP and composites, (**a**) Heating curves (**b**) Cooling curves.

Figure 4 and Table 3 present the melting and cooling curves, along with the relevant crystal data for each sample. Each sample exhibits two distinct melting peaks, situated around 128 °C and 163 °C, respectively. These melting peaks, denoted as T_m1_ and T_m2_, are expected to correspond to the β-crystal and α-crystal peaks of rPP, respectively. The formation of the β-crystal is linked to the presence of β-nucleating agents, shear forces, and temperature gradients during the crystallization process [21,22]. As Figure 4 depicts, despite CSP and SSP providing varying shear strengths, there is no significant alteration observed in T_m1_. This suggests that the genesis of the β-crystal in rPP is more likely influenced by impurities within the recycled material, with some of these impurities acting as β-nucleating agents.

Introducing rPET results in a reduction of both T_m1_ and T_m2_. For instance, in the case of rPP/rPET-CSP, T_m2_ is lowered by 1.5 °C when compared to that of rPP-CSP. This decrease may be attributed to the dispersed phases of rPET hindering the formation of a well-defined rPP crystalline structure. Moreover, the better the dispersibility of rPET, the more pronounced its inhibitory impact on rPP. Therefore, the incorporation of PP-g-Ma and the utilization of SSP can further decrease T_m2_, as evidenced by rPP/rPET/Ma-SSP, where T_m2_ is reduced by 2.9 °C in contrast to rPP-SSP.

The absence of cold crystallization peaks indicates that both rPP and rPET have fully crystallized after printing. The inclusion of rPET leads to a decrease in the crystallinity of rPP. Nevertheless, the introduction of PP-g-Ma serves to augment interfacial compatibility between rPET and rPP, providing additional nucleation sites. While an increased number of nucleation sites can potentially impede the formation of perfect crystal structures, they contribute to an overall enhancement of crystallinity. Moreover, the presence of rPET suppresses the crystallization of rPP, resulting in a shift of the crystallization peak from 122.5 °C in pure rPP-CSP to 120.0 °C in the PP/r-PET-CSP blend.

**Figure 5:**
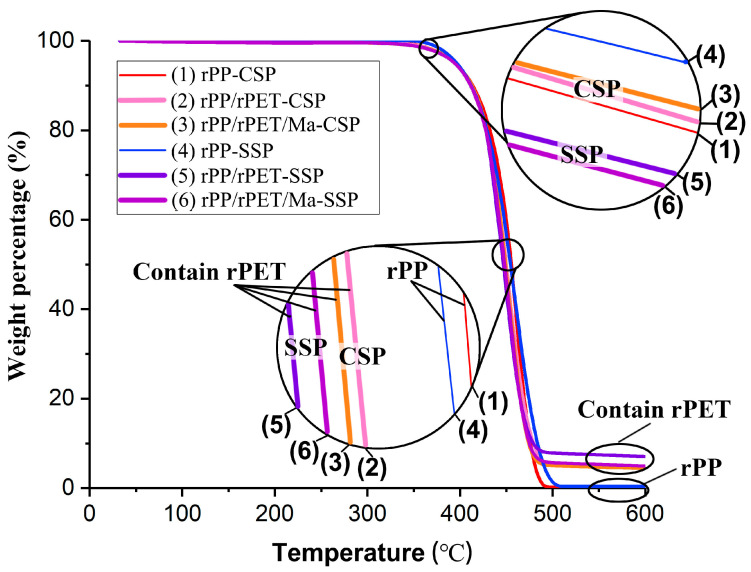
TGA curves of rPP and composites printed by CSP and SSP.

TGA is utilized to characterize the thermal decompositions of recycled polymers. Figure 5 plots the weight percentage (%) of each sample as a function of temperature. The initiation of specimen degradation occurs at approximately 350 °C. Notably, as observed in the enlarged graph, samples with rPET produced through CSP demonstrate higher initial degradation temperatures than those of SSP. This indicates that rPET exhibits higher thermal stability in CSP.

At the point of 50% thermal weight loss, rPP-SSP and rPP-CSP exhibit comparable thermal stability, suggesting that the residence time effect of SSP has a relatively minor influence on the degradation of rPP. However, the degradation curves for rPP/rPET-SSP and rPP/rPET/Ma-SSP are situated to the left of those for CSP. This indicates that the degradation in rPP/rPET is more pronounced in SSP compared to CSP.

This phenomenon can be attributed to the prolonged residence time and the pronounced shear effect of the SSP shear device. Reclaimed PET materials more often undergo partial deterioration due to thermal and shear degradation compared to virgin materials [23,24]. While, for SSP, the melt undergo a longer duration to traverse the heated barrel. Consequently, the rPP/rPET-SSP exhibits lower thermal stability. Furthermore, for samples with added rPET, residues are still present at 600 °C, likely due to inorganic impurities that may be present in the rPET.

**Figure 6:**
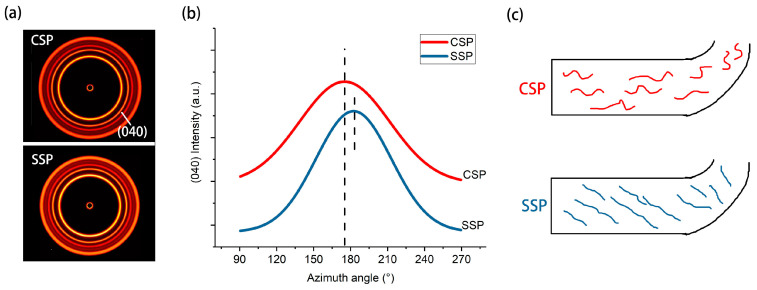
(**a**) 2D-WAXD patterns of CSP and SSP, (**b**) azimuthal profiles taken at the (040) α reflection as a function of azimuthal angle, and (**c**) schematical of oriented molecules in CSP and SSP.

To investigate the shearing impact of SSP on the extruded melt, Figure 6a presents the 2D-WAXD images of rPP-CSP and rPP-SSP. The azimuthal angle chart in Figure 6b is derived from the circular integration of the (040) crystal plane, providing insight into the degree of orientation and direction of rPP molecular chains. During the fused deposition modeling (FDM) printing process, the rPP melt undergoes both rotational shearing and drag forces. Rotational shearing encourages circumferential orientation of PP molecular chains around the screw, while the drag forces after extrusion align the chains axially. Consequently, the final orientation of the product results from the combined effects of these two mechanisms.

Both rPP-CSP and rPP-SSP encounter similar drag forces, but they exhibit peak azimuthal angles of 175° and 183°, respectively. This angular variance is a consequence of distinct circumferential shear strengths. Moreover, the calculated orientation degrees for rPP-CSP and rPP-SSP are 0.52 and 0.60, respectively, indicating that the oriented molecules generated by circumferential shear are preserved after extrusion. In Figure 6c, a molecular arrangement schematic, illustrates that CSP tends to align molecular chains parallel to the printing direction with relatively low orientation, while SSP orients the molecular chains at a specific angle to the printing direction with a higher degree of orientation.

It is anticipated that rPET in the composites should follow a similar orientation pattern. Because of rPET’s higher crystallization temperature in comparison to rPP, its molecular chains are more prone to freezing, facilitating the retention of orientation. Consequently, its orientation degree should be higher than that of rPP. Nevertheless, due to the overlapping diffraction patterns of rPET with those of rPP, it is not feasible to determine the corresponding orientation degree for rPET using 2D-WAXD.

**Figure 7:**
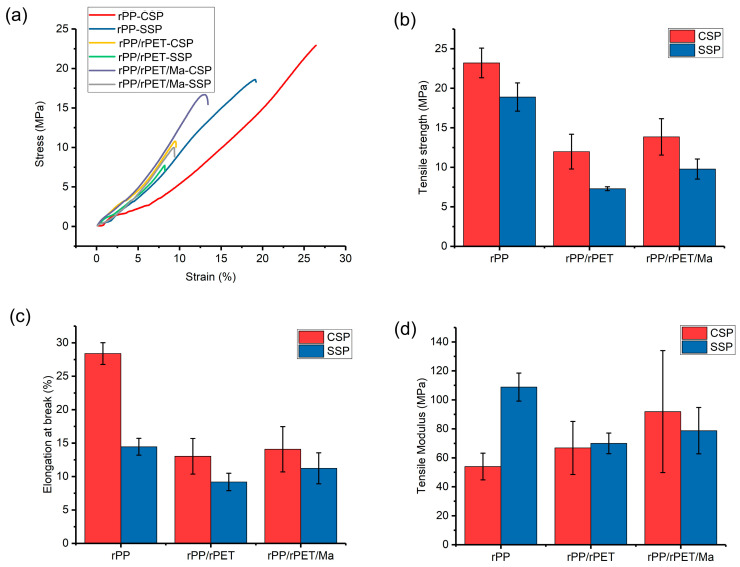
Tensile properties of rPP and its composite. (**a**) Selective stress−strain curves, (**b**) tensile strength, (**c**) elongation at break, and (**d**) tensile modulus.

Figure 7a displays the selective stress–strain curves, while Figure 7b presents the tensile strengths. rPP-CSP exhibits the highest tensile strength, reaching approximately 23 MPa. In contrast, SSP reduces the tensile strength of rPP-SSP to 19 MPa. This difference is primarily due to the printing direction set at 0°. In CSP samples, the molecular chains align parallel to the direction of the applied force. However, in SSP samples, the molecular chains are oriented at an angle to the applied force direction, making them more susceptible to chain slippage under stress, resulting in a reduction in tensile strength. Previous experiments have also indicated that while higher orientation enhances tensile strength in the direction of orientation, it can lead to reduced strength in other directions, causing the tensile strength of SSP samples to be lower than that of CSP samples [25].

rPET serves as a stress concentration point in rPP, and the poor interfacial compatibility between them results in a substantial reduction in tensile strength. The introduction of PP-g-Ma enhances interfacial compatibility and subsequently increases tensile strength. For instance, the tensile strength of rPP/rPET/Ma-SSP surpasses that of rPP/rPET-SSP by 34%.

rPP-CSP exhibits the highest elongation at the break, which is twice that of rPP-SSP. In the case of rPP-SSP, the direction of the force is not parallel to the orientation of the molecular chains, resulting in a reduced elongation at the break. Furthermore, SSP samples have a higher degree of orientation, and the oriented molecules are challenging to further elongate, as they exhibit greater rigidity. Consequently, rPP-SSP achieves the highest tensile modulus (109 MPa), a 102% increase compared to rPP-CSP (54 MPa). Additionally, due to the higher modulus of rPET compared to rPP, the addition of rPET contributes to an increase in the tensile modulus, while the improvement in interfacial compatibility resulting from PP-g-Ma further enhances the modulus.

Additionally, Figure 7 reveals that the CSP composites display a significantly larger error bar in various performances compared to the SSP composites. Excessive variation in sample performance can impede product utility. Therefore, it is imperative to delve deeper into the coefficient of variation (CV) for performance.

**Figure 8:**
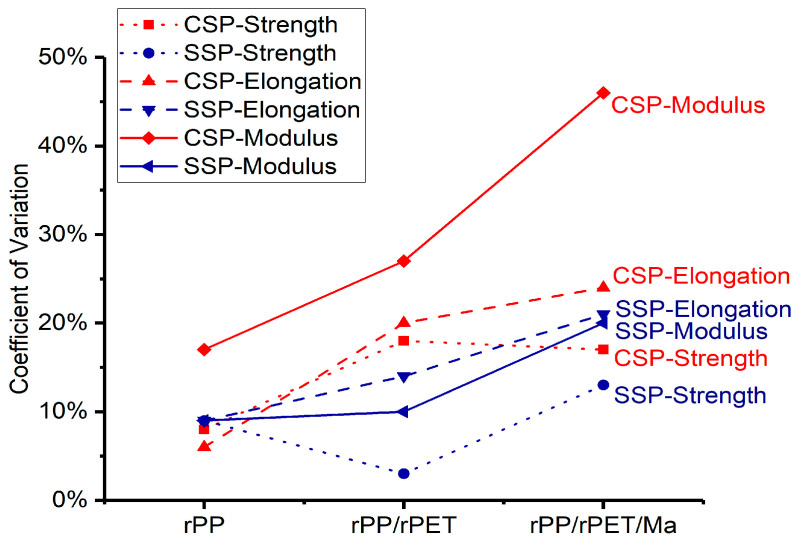
Coefficient of variation in strength, elongation, and modulus.

As Figure 8 depicts, the CV is represented by red and blue lines for CSP and SSP, respectively. In the case of rPP, except for CSP-Modulus, which exceeds 15%, the CV for all other performance parameters remains below 10%. However, the introduction of rPET leads to CV values exceeding 15% for all CSP’s tensile properties. In contrast, the CV values for SSP exhibit a milder increase, all remaining below 15%. The inclusion of PP-g-Ma results in an overall increase in CV values for all performance parameters, attributed to the lower PP-g-Ma content, which hinders the achievement of more uniform blending.

In general, for these blends, the SSP-CV of tensile properties are lower compared to CSP-CV. This phenomenon is attributed to the superior blending effect of SSP, facilitated by the high shear forces it provides. In CSP blends, the uneven dispersion of rPET and PP-Ma leads to significant fluctuations in mechanical performance. This is particularly noticeable in the case of the tensile modulus for rPP/rPET/Ma-CSP, where the CV approaches 50%, significantly impairing its utilization. Consequently, for multi-component recycled plastics, SSP offers a more stable tensile performance compared to CSP.

**Figure 9:**
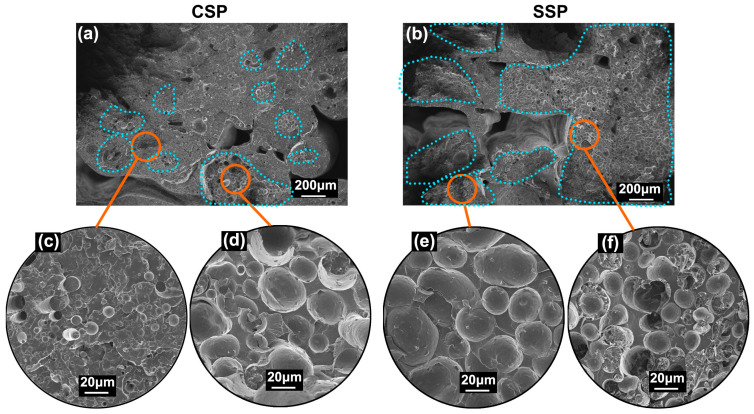
SEM image of the broken surface of (**a**) rPP/rPET-CSP and (**b**) rPP/rPET-SSP. Local magnification photos are shown in (**c**–**f**).

The SEM images of the tensile fracture surfaces offer a more visually compelling illustration of the disparities between CSP and SSP. Figure 9a,b depict the fracture surfaces of rPP/rPET-CSP and rPP/rPET-SSP, respectively. The region enclosed by the blue dashed lines contains dispersed rPET phases, with minimal rPET found outside this enclosed area.

In the magnified views of Figure 9c,d, the distribution of rPET within different zones of CSP samples exhibits significant non-uniformity, leading to notable fluctuations in extruded filament diameter and tensile performance. In contrast, the dashed areas in SSP samples encompass almost the entire cross-section (Figure 9b), and the magnified images reveal similar rPET dispersion (Figure 9e,f). This creates conditions for achieving more consistent melt extrusion diameter and tensile performance in SSP samples.

**Figure 10:**
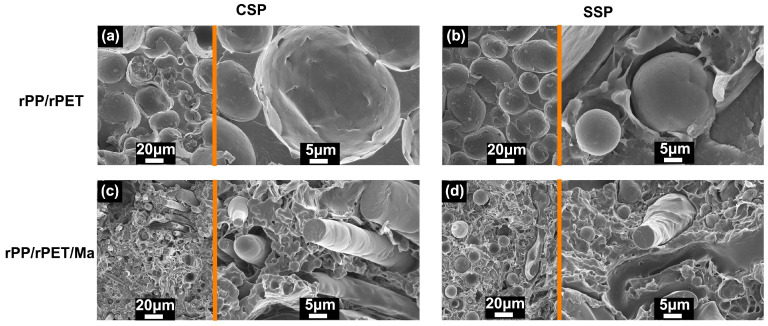
SEM image of the broken surface of (**a**) rPP/rPET-CSP and (**b**) rPP/rPET-SSP, (**c**) rPP/rPET/Ma-CSP, and (**d**) rPP/rPET/Ma-SSP.

Figure 10 illustrates the influence of PP-g-Ma on the morphology of rPET. As seen in Figure 10, rPP and rPET exhibit distinct, separate phases, indicating their complete immiscibility even in the presence of the compatibilizer. However, PP-g-Ma has a noticeable effect on the shape of rPET. In the absence of PP-g-Ma, rPET retains a spherical shape in both CSP and SSP (Figure 10a,b). With the incorporation of the compatibilizer, rPP/rPET/Ma-CSP reveals the presence of rPET fibers (Figure 10c), and these fibers are oriented perpendicular to the fracture surface. This arrangement is beneficial for enhancing the ability of rPET fibers to withstand greater external forces, resulting in improved tensile strength and modulus. Such composites are referred to as “in situ microfiber or microfibril composites”, known for their advantages, including reduced energy consumption, enhanced production efficiency, and decreased wear on processing machinery [26]. In contrast, rPP/rPET/Ma-SSP features fewer fibers, which can be attributed to the offset orientation in SSP samples. While rPET fibers are present, they are not oriented perpendicular to the interface, limiting their contribution to the enhancement of mechanical performance.

Comparing the various properties of CSP and SSP samples, as Table 4 indicates, CSP demonstrates strengths in terms of degradation resistance, increased tensile strength, and elongation at break. In contrast, SSP enhances dimensional stability, blending efficiency, tensile modulus, and the stability of tensile properties.

## 4. Conclusions

We employed self-developed CSP and SSP to directly 3D print recycled PP and PET blends from pellet materials. The high-shear device of SSP induced a substantial pressure drop, allowing for a more than a twofold increase in the printing screw speed compared to CSP. Along with the shear-induced blending effect, this led to a remarkable 50% reduction in the coefficient of variation (CV) for the extruded filament diameter compared to CSP. Furthermore, the effective blending effect of SSP enhanced the consistency of mechanical properties, maintaining consistently lower CV values than those of CSP samples. SSP’s high circumferential shear further increased the molecular chain orientation degree of rPET and rPP, altering its orientation relative to the melt’s extrusion direction. This chain orientation simultaneously improved the tensile modulus.

While SSP has the aforementioned advantages, the elevated pressure drops also extended the residence time of the melt within the barrel, resulting in partial degradation of the rPP/rPET blend and diminished thermal stability. This led to a decrease in tensile strength and elongation at break. This impedes the utilization of SSP in recycled rPP/rPET materials. While reducing the processing temperature is anticipated to mitigate the degree of degradation, it requires an increase in both the diameter and length of the screw to ensure comprehensive melting of the plastic. Further optimization of processing and screw parameters is essential to preserving the tensile strength of the blended recycled material.

## Figures and Tables

**Table 1:** Summary of mechanics design values for the screw extruder.

Screw’s external diameter (D)	16 mm
Aspect ratio L/D	15
Length of feeding, compression, and metering zones	4D, 6D, 5D
Fixed pitch	10 mm
Clearance to barrel	75 μm
Flight width	4 mm
Compression ratio	3.29

**Table 2:** Examples of blend composition and printing methods.

Sample	Printer	rPP (wt.%)	rPET (wt.%)	PP-g-Ma (wt.%)
rPP-CSP	CSP	100		
rPP/rPET-CSP	CSP	70	30	
rPP/rPET/Ma-SSP	SSP	60	30	10

**Table 3:** The melting temperature, crystal temperature, and crystallite.

Sample	T_m1_	T_m2_	T_c_	X_c_
rPP-CSP	129.7	163.8	122.5	31.4%
rPP/r-PET-CSP	128.2	162.3	120.0	26.5%
rPP/r-PET/Ma-CSP	127.1	161.9	120.5	31.9%
rPP-SSP	129.9	163.4	122.5	28.2%
rPP/r-PET-SSP	126.6	161.5	120.9	27.9%
rPP/r-PET/Ma-SSP	127.4	160.5	120.4	30.1%

**Table 4:** Advantages and disadvantages of CSP and SSP in printing recycled blends.

	Blending	Dimensional Stability	Tensile Stability	Anti- Degradation	Tensile Strength	Tensile Modulus	Elongation at Break
CSP				✔	✔		✔
SSP	✔	✔	✔			✔	

Note: Infill angle is 0°.

## Data Availability

Research data are contained within the article and may not be visible on other website.
